# Complex torcular dural arteriovenous fistula leading to cortical venous reflux-induced severe varix and subsequent bilateral cerebral hemispheric hemorrhage: a case report

**DOI:** 10.3389/fneur.2023.1303234

**Published:** 2023-12-14

**Authors:** Ziqi Li, Pengwei Hou, Qizuan Chen, Shuwen Mu, Jun Li, Yi Fang, Wenting Jiang, Xinhua Tian, Shousen Wang

**Affiliations:** ^1^Department of Neurosurgery, Fuzong Clinical Medical College of Fujian Medical University, Fuzhou, China; ^2^Department of Neurosurgery, Dongfang Affiliated Hospital of Xiamen University, School of Medicine, Xiamen University, Xiamen, China; ^3^Department of Neurosurgery, Peking Union Medical College Hospital, Chinese Academy of Medical Sciences, Peking Union Medical College, Beijing, China; ^4^Fujian Children’s Hospital (Fujian Branch of Shanghai Childre Medical Center), College of Clinical Medicine for Obstetrics Gynecology and Pediatrics, Fujian Medical University, Fuzhou, China; ^5^Department of Neurosurgery, The Affiliated Zhongshan Hospital of Xiamen University, Xiamen, China

**Keywords:** dural arteriovenous fistula, intracerebral hemorrhage, cortical venous reflux, superior sagittal sinus, venous compensation, venous variation

## Abstract

**Background and importance:**

Dural arteriovenous fistulas (dAVFs) with cortical venous reflux (CVR) are associated with a higher incidence of intracranial hemorrhage (ICH). We report a rare case of a complex torcular dAVF with severe cortical veins (CV) varix leading to extensive bilateral cerebral hemorrhages. This discovery suggests a potential new subtype of dAVF. The case underscores the necessity of a comprehensive understanding of hemodynamic changes in dAVFs and the importance of considering venous compensatory capacity in treatment. This case challenges existing classifications and treatment strategies for dAVFs, highlighting the need for further research and discussion within the neurosurgical community.

**Clinical presentation:**

A 56-year-old male was admitted to the hospital presenting with dizziness, fatigue, and numbness. Brain CT scans revealed extensive bilateral cerebral hemorrhages. Digital subtraction angiography (DSA) identified a complex torcular dAVF. No cerebral sinus venous thrombosis was detected, but a venous variation in the left transverse sinus was observed. Preoperative DSA demonstrated the patient’s well-developed venous compensatory ability. Subsequently, the patient underwent transarterial embolization. The patient made a good recovery. Follow-up DSA and MR angiography at 3 months and 1 year post-treatment showed no recurrence.

**Conclusion:**

DAVFs are rare lesions, prone to ICH, particularly when CVR is involved. We report a rare case of CVR with severe varix leading to hemorrhagic lesions in both cerebral hemispheres. Our aim is to alert neurosurgical colleagues worldwide to this potential new subtype and to evaluate treatment options, in order to assist those who may encounter such cases in the future.

## Background

Dural arteriovenous fistulas (dAVFs), abnormal shunts situated within the dura mater (1), constitute 10–15% of all intracranial vascular malformations (2). Predominantly affecting individuals aged between 50 and 60 (3), the clinical manifestations of dAVFs can vary, largely dependent on their location (4). When cortical venous reflux (CVR) is involved, the dAVF is considered high-grade, leading to an elevated risk of hemorrhage, increased surgical complexity, and a less favorable prognosis (3, 5–10).

Existing classifications primarily focus on the angiographic presentations of dAVFs, incorporating the adverse impacts of intracranial hemorrhage (ICH) and Non-Hemorrhagic Neurological Deficits (NHND) (3, 5, 6). However, these classifications may not fully capture the significance of CVR, potentially resulting in an underestimation of disease severity and influencing treatment decisions. Furthermore, the role of the intracranial venous system in dAVFs is often overlooked, despite its potential substantial influence on both etiology and prognosis (11). An effective preoperative evaluation of the venous system can significantly improve patient survival rates (12). In this report, we present a unique case of a dAVF leading to severe cortical venous varices, which resulted in widespread hemorrhagic lesions in both cerebral hemispheres due to CVR. We also discuss the valuable insights and implications derived from this case.

## Clinical presentation

**Figure 1:**
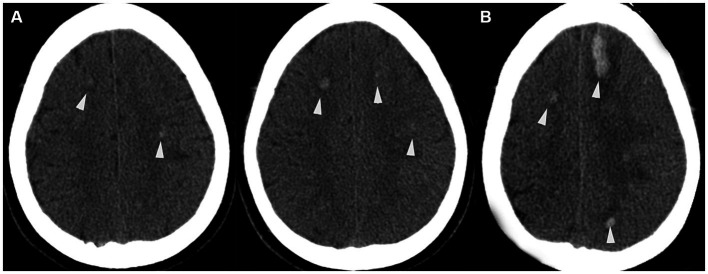
**(A)** Admission axial CT scan, indicating multiple scattered hemorrhagic foci in both cerebral hemispheres (white arrowheads). **(B)** Preoperative axial CT scan showing the progression of hemorrhage in the left frontal lobe (white arrowheads).

A 56-year-old male patient with a history of deep vein thrombosis (DVT) and chronic headaches, but no history of head trauma, surgery, or other conditions such as intracranial infection or hypertension, was admitted to the hospital due to dizziness, fatigue, and numbness. Neurological examination revealed clear consciousness and slightly weakened limb strength. Blood tests, including complete blood count and coagulation index, were completely normal (Table 1, Supplementary material). Ophthalmological examination revealed papilledema (Figure 1, Supplementary material). Brain CT scans showed multiple scattered hemorrhagic foci in both cerebral hemispheres. Digital subtraction angiography (DSA) of the cerebral vessels suggested an arteriovenous shunt around the torcular. The shunt was supplied by bilateral occipital arteries (OA), the left middle meningeal artery (MMA) occipital branch, and the vertebral artery (VA) meningeal branch, and drained into the superior sagittal sinus (SSS) and cortical veins (CV). No cerebral sinus venous thrombosis (CVST), but a venous variation in the left transverse sinus (TS) was found, impeding its normal venous drainage. The patient became somnolent the next day, with increased hemorrhage in the left frontal and parietal lobe. Upon further examination, it was observed that the cerebral hemorrhage had significantly intensified compared to the time of admission (Figure 1). An emergency plan was made to embolize the shunt arterially. Informed consent was obtained before treatment.

**Table 1:** Classification of dAVFs.

A. Borden classification and Cognard classification							
Natural course	Borden classification			Cognard classification			
	Type	Venous drainage site	CVR	Type	Venous drainage site	Flow pattern in sinus	CVR
Benign	I	Dural sinus	No	I	Dural sinus	Antegrade	No
Benign				IIa	Dural sinus	Retrograde	No
Aggressive	II	Dural sinus	Yes	IIb	Dural sinus	Antegrade	Yes
Aggressive				IIa + b	Dural sinus	Retrograde	Yes
Aggressive	III	Cortical vein	Yes	III	Cortical vein		Yes without venous ectasia
Aggressive				IV	Cortical vein		Yes with venous ectasia
Aggressive				V	Cortical vein with spinal medullary drainage		Yes

B. Modification to angiographic classification systems for DAVFs proposed by G. J. Zipfel et al.							
Modified type	Borden-Shucart Type	Cognard Type	Venous Drainage	CVD	Annual Risk (%)	Treatment Recommendation	
					ICH	Death	
1	I	I, IIa	dural sinus	No	<1	0	Elective treatment for intractable symptoms
2 w/aCVD	II	IIb, IIa + b	dural sinus	Yes	1.4–1.5	0	Elective treatment to prevent ICH/NHND
2 w/sCVD	II	IIb, IIa + b	dural sinus	Yes	7.4–7.6	3.8	Immediate treatment to prevent ICH/NHND
3 w/aCVD	III	III, IV, V	CVD	Yes	1.4–1.5	0	Elective treatment to prevent ICH/NHND
3 w/aCVD	III	III, IV, V	CVD	Yes	7.4–7.6	3.8	Immediate treatment to prevent ICH/NHND

CVR, cortical venous reflux; dAVF, dural arteriovenous fistula. CVD, cortical venous drainage; aCVD, asymptomatic CVD; sCVD, symptomatic CVD.

**Figure 2:**
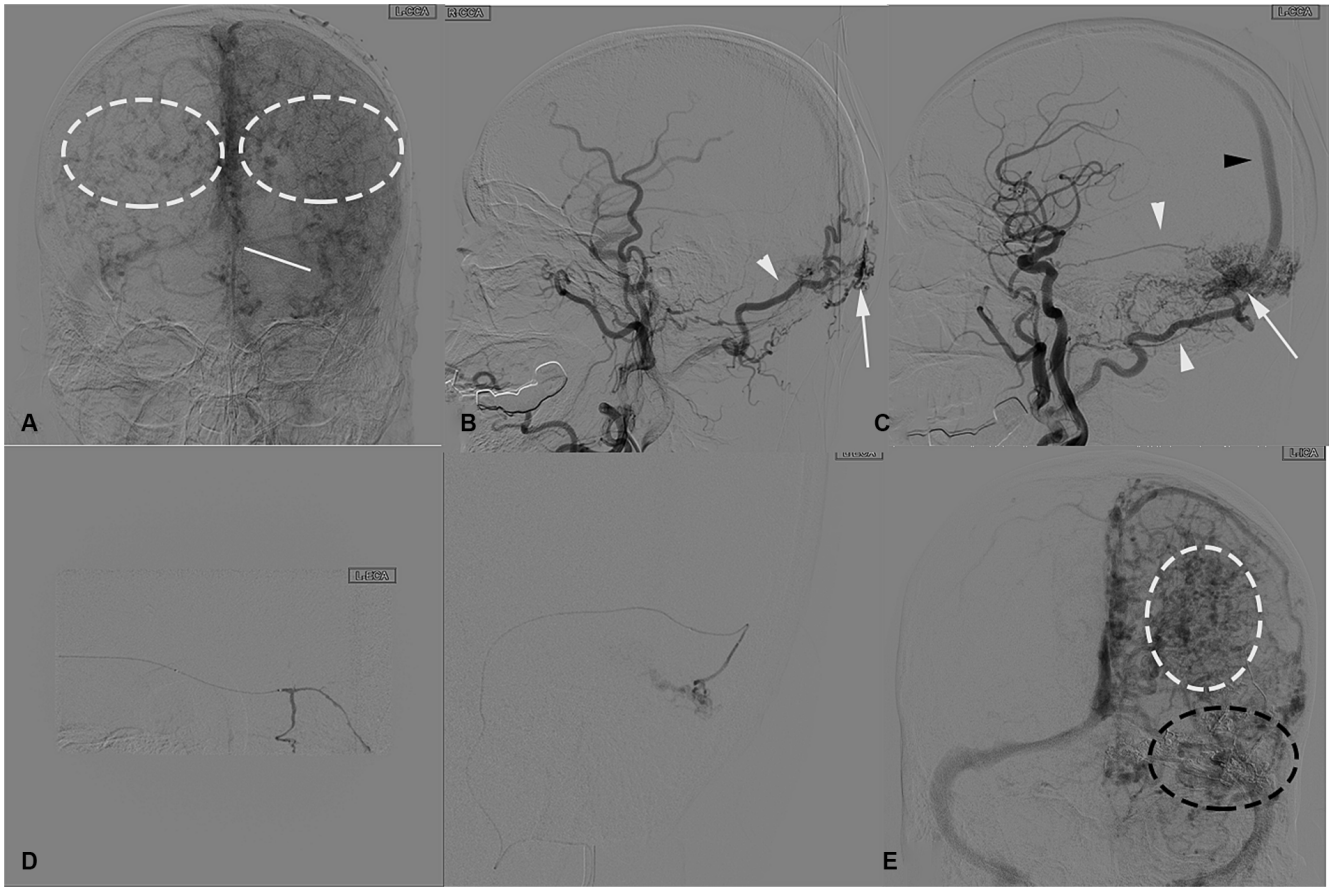
**(A)** Anteroposterior view of the left common carotid artery angiography venous phase before endovascular surgery, a congenital absence of the left transverse sinus is shown (white solid line) and severe varix of bilateral cortical veins (white dashed circles). In the lateral view of the left common carotid artery angiography, **(B,C)** demonstrating an arteriovenous shunt (white arrowheads). The arteriovenous shunt is fed by the left occipital artery (white arrow), left MMA occipital branch (white arrow), and drains into SSS (black arrow) around the torcular area. **(D)** Superselective angiography of the left occipital artery and MMA branch obtained before ONYX glue injection, demonstrating multiple arterial channels flowing into the fistula. **(E)** Intraoperative angiography obtained after ONYX injection shows a cast of glue occluding the fistula, including all the left feeding arteries and part of the compensatory veins (black dashed circle), with increased cortical vein varix (white dashed circle).

Under local anesthesia, a 6-Fr Envoy guiding catheter (Cordis) was placed in the internal carotid artery (ICA), and a Microcatheter (Hyperform 4 × 7 mm, America MTI/EV3) was navigated into the left MMA occipital branch and left OA branches. ONYX (Medtronic, Irvine, CA, United States.) glue was used for embolization of the dural arteriovenous fistula. After several attempts, the final angiography showed no branches supplying the fistula from the left internal and external carotid arteries, but a small amount of supply from the right OA remained, suggesting a slight residual shunt (Figure 2).

**Figure 3:**
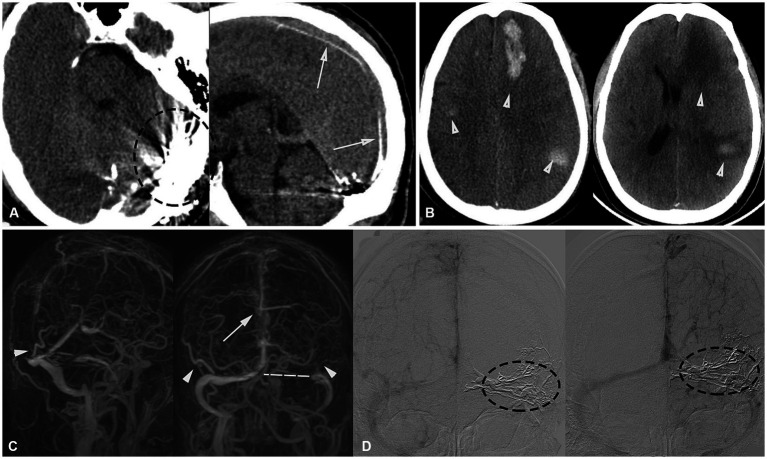
**(A)** Post-embolization axial and sagittal CT scans show a high-density area, demonstrating a cast of glue occluding the fistula (black dashed circle), including feeding arteries and the SSS (white arrow). **(B)** Postoperative follow-up CT revealed hemorrhage in the left frontal lobe, posterior temporal region, and right temporal lobe. Discharge review showed that the hematoma had been essentially absorbed (white arrowhead). **(C)** Postoperative follow-up MRV images in oblique posterior and posterior views revealed well-developed bilateral Labbé veins (white arrowheads), a variation in the left transverse sinus (white dashed line), and the presence of thrombosis in the SSS (white arrow). **(D)** DSA 3-month follow-up compared to post-surgery shows a significant reduction in cortical venous varix (left), with the fistula completely occluded and no recurrence.

Postoperative CT scanning revealed a high-density area indicating a cast of ONYX glue in the SSS. In the following week, intracranial hematoma continued to progress but eventually absorbed. Follow-up DSA 3 months after surgery demonstrated no recurrence of the dAVF and the varix in the bilateral cerebral hemispheres was markedly reduced. A one-year postoperative MR review indicated no recurrence of dAVF, although thrombosis was observed in the SSS (Figure 3), the patient’s condition and vision improved, with no significant neurological abnormalities.

## Discussion

Hemorrhage due to dAVF accumulation in the SSS is not uncommon, as documented in previous studies (13, 14). The distinguishing feature of this case, however, is the severe varix of the draining CV, leading to widespread hemorrhagic lesions in the bilateral cerebral hemispheres, a phenomenon rarely reported. This indirectly suggests an extraordinarily high pressure within the SSS, leading us to postulate that the blood within the SSS essentially has arterial characteristics, with a pressure and impact force equivalent to arterial blood. We term this phenomenon ‘venous arterialization’, which further exacerbates the risk of hemorrhagic events (15). To date, no clinical studies have proposed this concept.

The preoperative cerebral hemorrhage patterns observed in our patient warrant particular attention. The initial CT scan revealed multiple hemorrhagic foci across both cerebral hemispheres, predominantly attributable to the severe varix of the draining cortical veins. This finding underscores the significant impact of venous varicosities on cerebral hemorrhage. Subsequently, the second CT scan demonstrated an increased hemorrhage in the left frontal and parietal lobes. This progression can be directly ascribed to the elevated pressure in the SSS, indicative of a distinct hemorrhagic mechanism compared to the initial presentation. These observations highlight the complex interplay between venous features and hemorrhagic patterns, particularly in the context of venous arterialization and its hemodynamic consequences (16).

The etiology of dAVFs is multifaceted, encompassing trauma, surgery, venous stenosis, or CVST (17). In this case, DSA revealed an anatomical variation in the torcular area, a variation that accounts for about 8% of transverse sinus anomalies (18), leading to the absence of the left transverse sinus in this patient. We postulate that this could be a pivotal factor in the formation of the dAVF in this case. Previous studies have provided evidence that congenital anomalies in the venous system’s anatomy may be associated with the occurrence of intracranial vascular variations (19–21). However, this perspective is not commonly reported in cases of dural arteriovenous fistulas.

In this case, we observed a significant increase in pressure in the SSS due to ‘venous arterialization’. We hypothesize that this could be associated with the specific anatomical location of the lesion, the complex and diverse arterial supply to this area, the relatively singular venous drainage outlet, and the patient’s own venous variation. These factors may collectively contribute to a significant alteration in hemodynamics (16).

The patient’s bilateral inferior anastomotic veins, both cavernous sinuses, and the basal sinus were notably well-developed. MR imaging further revealed that the left CV connected to the superior and inferior petrosal sinuses via the cavernous sinus, ultimately draining into the sigmoid sinus, underscoring a pronounced venous compensatory capacity. This capacity largely determined the favorable postoperative prognosis. From embryonic development to adulthood, some veins degenerate during growth (18), but this does not imply a complete loss of function in adulthood. Under certain pathological conditions, some degenerated veins may be reactivated (22, 23). Through intraoperative DSA observation, we have reason to conclude that some collateral venous circulation in this patient has been reopened. This finding suggests that we should pay more attention to the importance of intracranial veins in the management of dAVFs.

In the current case of dAVF, the transarterial approach was unequivocally the superior choice, aligning with existing guidelines (24). Prior to embolization, an attempt was made to access the contralateral sigmoid-transverse sinus, which proved unsuccessful. The dAVF was primarily supplied by bilateral OA, the left MMA occipital branch, and the VA meningeal branch. In contrast, the venous drainage was predominantly through the SSS, which was under high pressure. The transarterial approach offered several advantages, including reduced risk of redirecting blood flow to alternative venous pathways, preservation of functional venous systems, and minimization of complications unique to the transvenous route, such as abducens nerve palsy (25). Before the Onyx era, transvenous approach was the mainstay of endovascular treatment for cure of dAVF. Modern transvenous techniques involve retrograde catheterization of the affected sinus and cortical veins, using microcoils, liquid embolic agents, or a combination for occlusion (26). However, the transvenous approach necessitates meticulous patient selection to achieve complete occlusion and avoid complications. Given the complex arterial supply and singular, high-pressure venous drainage in this case, the transarterial route was indeed the most judicious choice.

The shift from using n-BCA to Onyx as the embolic agent of choice in dAVF treatment is indicative of evolving clinical perspectives, driven by the quest for better control and reduced complications. While n-BCA has been a staple for high-flow dAVFs, its limitations, such as reduced controllability and a higher risk of complications, have made Onyx a more favorable choice (27). Onyx’s longer injection time allows for a more controlled embolization, particularly beneficial when venous routes are compromised (28). This case employed Onyx 20, aligning with its established safety profile and efficacy. However, it’s crucial to acknowledge that even with the optimal choice of Onyx and a transarterial approach, we observed some unintended glue migration into the SSS in postoperative imaging. This serves as a cautionary note on the inherent complexities and risks involved, emphasizing the need for meticulous technique and material selection. The newer agents like Squid and PHIL, although promising, are yet to be widely adopted in clinical practice (29, 30).

According to the prevalent classifications (6, 7) (Table 1), this case falls under Cognard Classification IV or Borden classification III. It is crucial to recognize the historical significance of the DjinDjian classification (31) in laying the groundwork for our understanding of cranial AVFs, which has substantially influenced subsequent classifications, including those referenced in our study. Transarterial embolization remains the preferred treatment for this type (24). Although Zipfel et al. (5). have expanded the classification to account for ICH and NHND, our case presents unique challenges to these existing frameworks. The severe cortical venous varicosities in our patient, leading to bilateral widespread scattered hemorrhages, highlight the limitations of current classifications and suggest the need for their refinement. Future classifications could benefit from differentiating between unilateral and bilateral hemorrhages, as well as incorporating a grading system for venous ectasia severity. Additionally, considering venous anomalies in the classification could provide a more comprehensive understanding of these conditions and their implications for treatment. Furthermore, integrating a post-embolization therapeutic classification could offer valuable insights into the outcomes and efficacy of interventional treatments, enhancing our approach to managing these complex cases. Unlike merely indicating the presence of CVR or ICH, the occurrence of these kinds of hemorrhages implies higher intracranial pressure, which poses a challenge to our treatment choices, necessitating a careful evaluation of the patient’s venous compensatory ability before determining the final approach.

## Conclusion

This case study presents a rare instance of a complex torcular dural arteriovenous fistula leading to severe cortical venous reflux-induced varix and subsequent bilateral cerebral hemispheric hemorrhage. It offers novel insights into the etiology, hemodynamics, classification, and venous compensation of this condition. The case underscores the need for further research and a broader understanding of these aspects, emphasizing the importance of considering venous compensatory capacity in treatment planning. It challenges existing classifications and calls for more attention to this potential subtype in the neurosurgical community.

### Patient perspective

“I first came to the hospital’s neurosurgery outpatient clinic because I was feeling dizzy and tired. The doctors ran a bunch of tests, including a digital subtraction angiography (DSA), which showed something wasn’t right with the blood vessels in my brain. They moved me quickly to the ICU, and I remember being aware of what was happening. But by the next day, things started to get really uncomfortable, and everything around me seemed blurry and confusing.

At the time, I did not understand why I was feeling that way. It was only later that I learned from my family that the doctors had found my bleeding was getting worse, and that’s why they rushed me into surgery. I do not recall much about the procedure itself, just waking up to anxious faces and the beeping of machines. After the surgery, I spent a week in the ICU, which was filled with pain and uncertainty, and I had to rely on my family and doctors to piece together what happened during those days.

As time went on, the doctors told me and my family that my bleeding was under control and the hematoma in my brain was slowly being absorbed. That period was a rollercoaster of emotions, from despair to hope. Follow-up checks 3 months later showed that my arteriovenous fistula had not come back, and the varices in my brain were much better. Even though a check-up a year later found a clot in my superior sagittal sinus, the doctors said it did not need any special treatment.

Now, I can pretty much take care of myself. I get the occasional slight headache, but there are no serious neurological issues, and my vision has improved. Looking back, that time was as much a challenge as it was a period of growth for me. I’m deeply grateful for the skill and care of the doctors and nurses. They did not just treat my condition; they gave me a chance at a new life”.

### Limitations

A limitation of this case is the missing ophthalmological imaging from an outpatient visit. Only written records are available. Nonetheless, follow-up imaging adequately addresses this inconvenience.

## Data availability statement

The raw data supporting the conclusions of this article will be made available by the authors, without undue reservation.

## Ethics statement

Written informed consent for the publication of any potentially identifiable images or data included in this article was obtained from the individual(s) and their next of kin.

## Author contributions

ZL: Conceptualization, Data curation, Formal analysis, Investigation, Methodology, Software, Writing – original draft. PH: Conceptualization, Software, Visualization, Writing – original draft. QC: Conceptualization, Project administration, Supervision, Writing – original draft. SM: Data curation, Formal analysis, Writing – original draft. JL: Conceptualization, Supervision, Writing – original draft. YF: Methodology, Writing – original draft. WJ: Investigation, Writing – original draft. XT: Project administration, Supervision, Writing – review & editing. SW: Conceptualization, Funding acquisition, Project administration, Resources, Supervision, Writing – review & editing.
