# Supplementary material for: Complex torcular dural arteriovenous fistula leading to cortical venous reflux-induced severe varix and subsequent bilateral cerebral hemispheric hemorrhage: a case report
Source: Front Neurol. 2023 Dec 14;14:1303234. doi: 10.3389/fneur.2023.1303234 (PMC10757922; doi:10.3389/fneur.2023.1303234)

**Supplemental Digital Content**

**Table 1. Blood tests, Coagulation index and Biochemical examination at admission**

**Figure1.** **Ophthalmology consultation records**

**Table 1. Blood tests, Coagulation index and Biochemical examination at admission.**

|  | result | Normal reference value | Single position |
| --- | --- | --- | --- |
| White blood cell count | 6.43 | 3.5-9.5 | 10^9/L |
| Granulocyte percentage | 70.1 | 40-75 | % |
| Granulocyte count | 4.51 | 1.8-6.3 | 10^9/L |
| Lymphocyte percentage | 23.6 | 20-50 | % |
| Lymphocyte count | 1.52 | 1.1-3.2 | 10^9/L |
| Monocyte percentage | 5.3 | 3-10 | % |
| Monocyte count | 0.34 | 0.1-0.6 | 10^9/L |
| Percentage of eosinophilic cells | 0.8 | 0.4-8 | % |
| The number of eosinophils | 0.05 | 0.02-0.52 | 10^9/L |
| Percentage of basophils | 0.2 | 0-1 | % |
| Number of basophils | 0.01 | 0.0-0.06 | 10^9/L |
| red blood cell | 5.09 | 4.3-5.8 | 10^12/L |
| Hemoglobin assay | 157 | 130-175 | g/L |
| Mean erythrocyte volume | 87.2 | 82-100 | fL |
| Hematocrit determination | 44.4 | 40-50 | % |
| Erythrocyte distribution width | 14 | 11.5-14.8 | % |
| Mean erythrocyte hemoglobin content | 30.8 | 27-34 | pg |
| Mean erythrocyte hemoglobin concentration | 354 | 316-354 | g/L |
| Platelet count | 121 | 125-350 | 10^9/L |
| Mean platelet volume | 9.9 | 6.0-11.5 | fL |
| Platelet accumulation | 0.12 | 0.11-0.28 | % |
| Platelet distribution width | 11.6 | 8.4-16.1 | % |

|  | result | Normal reference value | Single position |
| --- | --- | --- | --- |
| Fibrin (fibrinogen) degradation products | 2.2 | <5 | mg/L |
| D-dimer | 0.4 | <0.5 | mg/L FEU |
| PT | 12.1 | 9.8-12.1 | sec |
| PT-INR | 1.06 | 0.82-1.15 |  |
| APTT | 25.9 | 25-31.3 | sec |
| TT | 19.8 | 14-21 | sec |
| FIB | 2.76 | 2-4 | g/L |

|  | result | Normal reference value | Single position | |
| --- | --- | --- | --- | --- |
| Urea nitrogen (urease ultraviolet method) | 4.3 | 2.9-8.2 | | mmol/L |
| Creatinine(colorimetric) | 92 | 53-115 | | μmol/L |
| Total protein (biuret method) | 69..3 | 65-85 | | g/L |
| Albumin (bromocresol green method) | 42.4 | 40-55 | | g/L |
| globulin | 26.9 | 23-36 | | g/L |
| White sphere ratio | 1.58 | >1.1 | |  |
| Alanine aminotransferase (IFCC method) | 42.6 | 7-50 | | U/L |
| Alkaline phosphatase(AMP method) | 54.1 | 35-135 | | U/L |
| Glutamine transdermal enzyme (IFCC method) | 33.3 | 7-60 | | U/L |
| Total bilirubin (diazo method) | 9.4 | <21 | | μmol/L |
| Direct bilirubin (diazo method) | 2.2 | <5 | | μmol/L |
| Indirect bilirubin | 7.2 | <16 | | μmol/L |
| Creatine phosphokinase (UV method) | 100.4 | 26-174 | | U/L |
| Creatine kinase isoenzyme (inhibition method) | 7 | <25 | | U/L |
| Aspartate aminotransferase (IFCC method) | 32.2 | 13-40 | | U/L |
| Lactate degassing enzyme (L-P method) | 186 | 109-245 | | U/L |
| Cholesterol(Cholesterol oxidase method) | 3.18 | <5.2 | | mmol/L |
| Glycerin triacetate (GPO-PAP method) | 1.18 | <1.7 | | mmol/L |
| Low density lipoprotein (elimination) | 2.08 | <3.12 | | mmol/L |
| Sodium (indirect ISE method) | 137.4 | 137-147 | | mmol/L |
| chlorine (indirect ISE method) | 105.1 | 99-110 | | mmol/L |
| Calcium (colorimetric method) | 2.32 | 2.08--2.6 | | mmol/L |
| Magnesium (dimethvlaniline blue method) | 0.86 | 0.7-1.1 | | mmol/L |
| Phosphorus (molybdate ultraviolet method) | 1.04 | 0.9-1.34 | | mmol/L |
| Carbon dioxide(PEPC method) | 24.3 | 23-29 | | mmol/L |
| Osmotic pressure | 296.9 | 280-310 | | mmol/L |

**Figure 1. Ophthalmology consultation records.**


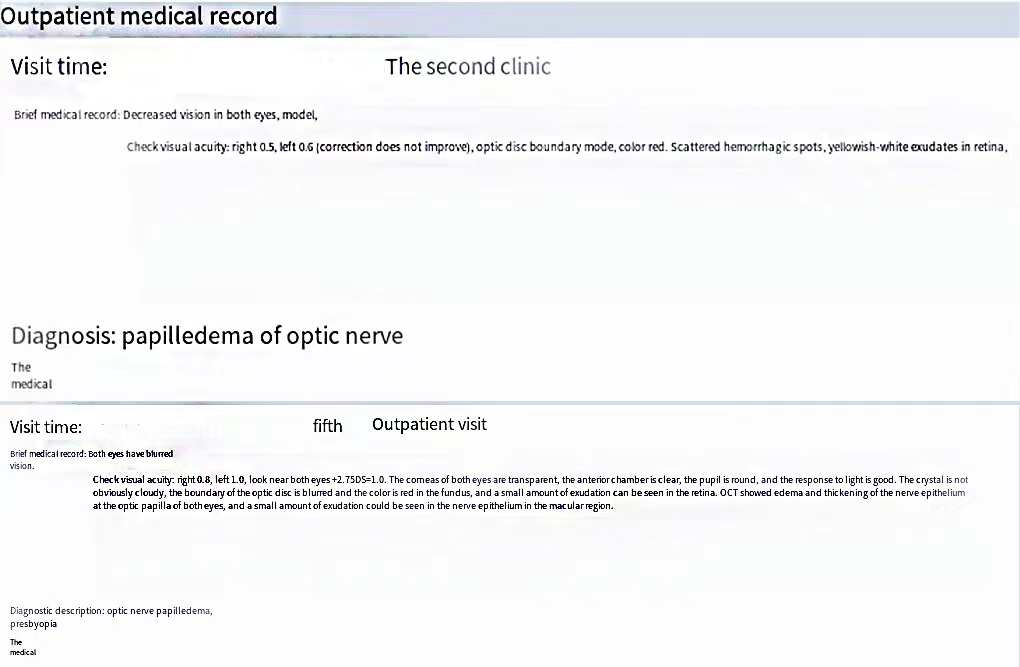

Supplement: Supplementary file 1 [file Data_Sheet_1.docx]
